# The Role of Glial Cells and Synapse Loss in Mouse Models of Alzheimer’s Disease

**DOI:** 10.3389/fncel.2018.00473

**Published:** 2018-12-11

**Authors:** Stephanie Ziegler-Waldkirch, Melanie Meyer-Luehmann

**Affiliations:** ^1^Department of Neurology, Medical Center—University of Freiburg, Freiburg, Germany; ^2^Faculty of Medicine, University of Freiburg, Freiburg, Germany

**Keywords:** Alzheimer’s disease, amyloid plaques, glial cells, synapse loss, microglia, astrocytes

## Abstract

Synapse loss has detrimental effects on cellular communication, leading to network disruptions within the central nervous system (CNS) such as in Alzheimer’s disease (AD). AD is characterized by a progressive decline of memory function, cognition, neuronal and synapse loss. The two main neuropathological hallmarks are amyloid-β (Aβ) plaques and neurofibrillary tangles. In the brain of AD patients and in mouse models of AD several morphological and functional changes, such as microgliosis and astrogliosis around Aβ plaques, as well as dendritic and synaptic alterations, are associated with these lesions. In this review article, we will summarize the current literature on synapse loss in mouse models of AD and discuss current and prospective treatments for AD.

## Synapse Loss in Neurodegeneration

Synapse loss has harmful effects on cellular communication, leading to network disruption in the central nervous system (CNS). The communication of billions of neurons within the mammalian brain generates and controls memory, thoughts and emotions. In a neuronal network with different cells, the transfer of information is coordinated at specialized compartments such as the synapse. Synapses are contact points between two neurons, where they communicate by passing ions or neurotransmitter across the synaptic cleft. Synapses can have excitatory or inhibitory effects on the target cells, depending on the released signals. The formed synapses are not rigid but rather dynamic and can either strengthen, shrink or even get lost. Considering the critical role of synapses under physiological conditions, it is not surprising that a severe loss of synaptic integrity can cause substantial disorders such as neurodegenerative diseases (Dudai and Morris, 2013).

Neurodegenerative diseases are disorders of the CNS or the peripheral nervous system characterized by the progressive structural and functional degeneration of neurons, leading to mental or movement problems. The most common form of neurodegenerative diseases is Alzheimer’s disease (AD) which currently affects 46 million people worldwide (Prince, 2015). Over a century ago Alois Alzheimer first described the defining lesions (Stelzmann et al., 1995), the two main hallmarks of AD, extracellular amyloid-β (Aβ) plaques and intraneuronal aggregates of hyperphosphorylated tau protein, so-called neurofibrillary tangles. Aβ is released from the amyloid precursor protein (APP) by cleavage of β- and γ-secretases (Haass, 2004) and accumulates in the extracellular space of the brain to diffuse or dense-core plaques (Serrano-Pozo et al., 2011). Intravital imaging studies of APP transgenic mice confirmed that smaller dense-core plaques can cluster together, thus forming lager plaques (McCarter et al., 2013) that are associated with neuronal and synapse loss (Tsai et al., 2004; Spires et al., 2005), increased neurite curvature (Garcia-Alloza et al., 2006; Meyer-Luehmann et al., 2008), impaired neuronal activity in dendritic segments (Meyer-Luehmann et al., 2009), dystrophic neurites (D’Amore et al., 2003; Tsai et al., 2004) and the accumulation of glial cells (Bolmont et al., 2008; Meyer-Luehmann et al., 2008; Kuchibhotla et al., 2009; Delekate et al., 2014). However, memory impairments and cognitive decline are most likely caused by synapse dysfunction and synapse loss rather than due to mere neuronal loss or the accumulation of Aβ plaques and neurofibrillary tangles (Terry et al., 1991; Masliah et al., 1994; Koffie et al., 2011). Electron microscopy and immunohistochemical stainings for synaptic markers revealed significant reductions in synaptic density in the cortex and hippocampus (Scheff et al., 1990; Terry et al., 1991; Masliah, 2001). Although the cause of synapse loss has not yet been fully elucidated, most likely both lesions, Aβ and tau, contribute to neurodegeneration.

Besides aging, new genetic risk factors for AD were reported recently in GWAS, such as ApoJ/Clusterin, PICALM, complement receptor 1 (CR1), TREM2 and sialic-binding immunoglobulin (Ig)-like lectin CD33 (Lambert et al., 2009; Naj et al., 2011; Hollingworth et al., 2012; Guerreiro et al., 2013; Jonsson et al., 2013). Interestingly, some of these genes are involved in Aβ production or clearance (Harold et al., 2009; Lambert et al., 2009), or are part of immune-related pathways. During development, synapse elimination was shown to be dependent on microglia phagocytosis that was mediated by C1q and C3 (Stevens et al., 2007). Recently, it was also demonstrated that in young pre-depositing hAPP mice this “developmental synaptic pruning pathway” is activated and leads to synapse loss (Hong et al., 2016).

Aβ plaque formation follows a nucleation-dependent polymerization, where monomers form dimers, oligomers, protofibrils and amyloid fibrils (Harper and Lansbury, 1997; Kumar and Walter, 2011). Aβ peptides are 36–43 amino acids in length, whereas Aβ42 is the most neurotoxic fragment, with the highest affinity to aggregate and represents the main component of senile Aβ plaques. Soluble Aβ oligomers are the most neurotoxic species that have been shown to impair long-term potentiation (LTP) (Walsh et al., 2002; Shankar et al., 2008) and enhance long-term depression (LTD) (Li et al., 2009), resulting in weakening of synapses. LTP has been related to the formation of new dendritic spines, increases of postsynaptic densities and the enlargement of spine heads (Maletic-Savatic et al., 1999; Nägerl et al., 2004). In contrast, LTD has been associated with spine shrinkage and loss (Nägerl et al., 2004). Other studies reported that the non-fibrillar forms of Aβ can affect learned behaviors in rodents (Cleary et al., 2005; Lesné et al., 2006; Freir et al., 2011). Recently, it was demonstrated that lower molecular weight oligomers are highly bioactive molecules that inhibit synaptic plasticity, alter cell-surface receptor levels and induce microglial inflammatory response (Yang et al., 2017). Soluble oligomers extracted from AD brains disrupt LTP and synaptic function *in vitro* and impair cognition when injected into healthy mice *in vivo* (Walsh et al., 2002; Cleary et al., 2005; Shankar et al., 2007). *In vivo* imaging studies revealed a loss of dendritic spines around plaques as a result of altered structural plasticity (Spires et al., 2005), whereas increased spine density and synaptic markers were obtained upon the removal of soluble oligomers (Spires-Jones et al., 2009). Together, these results support the idea that soluble forms of Aβ are toxic to synapses.

In mouse models of AD, synapse loss is primarily found around dense-core Aβ plaques (Koffie et al., 2009), whereas no synapses are lost in the vicinity of diffuse plaques (Masliah et al., 1990), thus indicating that dense-core Aβ plaques release toxic soluble Aβ oligomers into the surrounding tissue (Takahashi et al., 2004; Koffie et al., 2009), leading first to synaptic dysfunction and finally to complete synapse loss. In several mouse models of AD, synapse numbers are significantly decreased compared to non-transgenic control mice already at pre-depositing stages (Hsia et al., 1999; Mucke et al., 2000; Shankar et al., 2009; Harris et al., 2010).

The role of tau in synapse loss is less well established. During the course of AD tau gets hyperphosphorylated and accumulates in the somata and dendrites of neurons (Grundke-Iqbal et al., 1986). The intracellular aggregates of hyperphosphorylated tau form inclusions and neuropil threads, both of which are strongly related to neuronal apoptosis (Spires-Jones et al., 2009). In human AD brains and in mouse models of tauopathy, tangle bearing neurons comprise fewer synapses onto their somata and express less synaptic proteins compared to healthy neurons (Callahan et al., 1999; Ginsberg et al., 2000). The overexpression of mutant P301L in rTg4510 mice led to altered synaptic function and synapse loss (Crimins et al., 2011).

## Glial Cells

Neuronal synapse formation is based on the interplay between neurons and glial cells. Microglia, the immune cells of the brain parenchyma, regulate synapse formation (Parkhurst et al., 2013) and synapse engulfment via the complement system, which is part of the innate immune system (Wu et al., 2015). In contrast, astrocytes provide nutrients to neurons, take up and release neurotransmitters and provide structural support for neurons (Verkhratsky et al., 2010; Clarke and Barres, 2013). Oligodendrocytes are myelin-forming cells guaranteeing a fast movement of action potentials through axons. Recently, a new cell population was defined as oligodendrocyte precursor cells or NG2-glia (Dimou and Gallo, 2015). In the hippocampus synaptic transmission occurs between NG2-glia and axons. Furthermore, NG2-glia can receive direct excitatory and inhibitory synaptic input from neurons mediated by the neurotransmitters glutamate and GABA. However, the functional role of this neuron to glia synapse is not yet entirely understood (Lin and Bergles, 2004; Bergles et al., 2010). The discovery of NG2-neuron synapses offers the possibility to further investigate the relationship between NG2-glia and neurons in the brain. Interestingly, during their differentiation step from NG2-glia to more mature stages (oligodendrocytes), these cells lose their synapses with neurons (De Biase et al., 2010). Due to the dearth of data, we will focus in this review more on the role of microglia and astrocytes and synapse loss.

## Microglia

Microglia mediated synapse loss, or synapse pruning is an important physiological process for proper brain maturation during development. Understanding microglia function in healthy conditions can further help to get insights into their contribution to synapse loss and dysfunction early in disease. Microglia constantly extend and retract their processes and scan their local environment, thereby exploring the entire brain volume (Nimmerjahn et al., 2005). Several studies confirmed that microglia directly contact synaptic elements, thus affecting many synapses (Tremblay et al., 2010; Paolicelli et al., 2011; Schafer et al., 2012). Recent work has also shown that disruption of microglia function resulted in deficient synaptic pruning that was associated with weak synaptic transmission leading to functional connectivity deficits (Paolicelli et al., 2011; Zhan et al., 2014). Furthermore, this microglia-mediated synaptic elimination was shown to be dependent on neuronal activity (Schafer et al., 2012). In addition, depletion of microglia led to a reduction in motor-learning-dependent synapse formation (Parkhurst et al., 2013), implicating microglia in sculpting synaptic connectivity.

**Figure 1:**
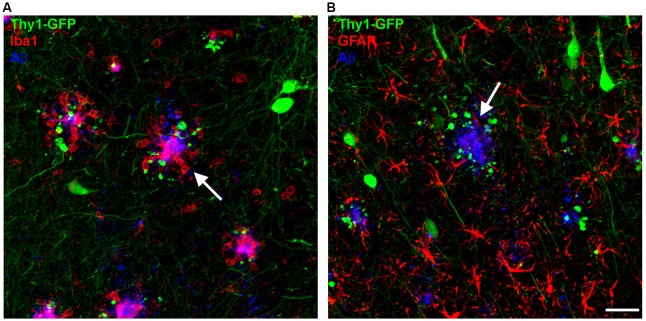
Microglia and astrocytes cluster around amyloid-β (Aβ) plaques (white arrows) in the brains of amyloid precursor protein (APP) transgenic mice. **(A)** Microglia (Iba1, red) can be found closely associated with Aβ plaques (6E10, blue), with dystrophic neurites appearing in the vicinity of Aβ plaques (GFP). **(B)** Reactive astrocytes (GFAP, red) can be found in close proximity to Aβ plaques (6E10, blue). Scale bar represents 10 μm.

Aβ plaques in human AD brains and in mouse models of AD are surrounded by microglia (Meyer-Luehmann et al., 2008; Serrano-Pozo et al., 2013) with impaired process extension (Figure 1A). Microglia cells can be classified into three main types based on their morphology: ramified, hypertrophic and amoeboid. Ramified microglia are found in plaque-free areas of the brain, whereas hypertrophic and amoeboid microglia with short, thick and poorly ramified processes are typically associated with senile plaques (Brawek et al., 2014). Interestingly, microglia are not only the resident monocytes in the brain but are also present in the retina, where Aβ deposits have been reported as well in AD patients and AD mice (Ning et al., 2008; Grimaldi et al., 2018). Similar to the brain, the retina of late-symptomatic AD mice contains less ramified microglia when compared to wildtype (WT) controls (Grimaldi et al., 2018). Recently, with the help of advanced technologies, more microglial phenotypes have been described. By comparing microglia cells from WT and 5xFAD transgenic mice using single-cell RNA-sequencing, disease associated microglia (DAM) co-localizing with Aβ plaques were identified (Keren-Shaul et al., 2017). Though, their precise role in synapse clearance and remodeling requires further investigation (Deczkowska et al., 2018). Moreover, an electron microscopy study defined “dark” microglia that are under steady state conditions rarely present but become prevalent in mouse models with AD pathology. Those “dark” microglia are predominantly active at synapses with condensed, electron-dense cytoplasm and nucleoplasm (Bisht et al., 2016). Ultimately, another study depicted the switch of microglia from a homeostatic to a neurodegenerative phenotype by gene expression analyses (Krasemann et al., 2017). However, the exact function of microglia in the context of AD is still not understood. In any case, they play either a beneficial or detrimental role in AD pathology, including the degradation of Aβ or the stimulation of neurotoxicity through inflammatory cytokine release (Wyss-Coray and Rogers, 2012). Several genes expressed or enriched in microglia appeared to be involved in Aβ clearance, including CD33 (Griciuc et al., 2013). Furthermore, members of the classical-complement-cascade, Clusterin and CR1 have been linked to late onset AD (Jun et al., 2010; Fonseca et al., 2016). The best characterized molecules involved in synapse removal by microglia are components of the complement cascade that is upregulated in AD brains. Furthermore, Aβ and tau aggregates can induce microglial and complement activation (Rogers et al., 1992; Shen et al., 2001). A recent study implicates microglia, complement and immune-related pathways as early mediators of synaptic dysfunction (Hong et al., 2016). In the hippocampus of AD mice, the complement proteins C1q and C3 were upregulated and connected with synapses at pre-depositing stages, causing extended engulfment of synaptic elements (Hong et al., 2016). Furthermore, inhibition of C1q, C3 and CR3 rescued synapse loss and synaptic dysfunction in young hAPP mice indicating that microglia are involved in early synapse loss in pre-depositing mice. In addition, C1q-deficient mice that were crossed to Tg2576 mice displayed less astrogliosis and Aβ plaques, suggesting a detrimental role of the complement pathway (Fonseca et al., 2004). Together, these data indicate that pathways responsible for synaptic pruning during development are activated in AD that eventually lead to synapse loss (Stephan et al., 2012; Hong et al., 2016). Interestingly, depletion of microglia (30%) in 3xTg AD mice improved cognition but did not alter Aβ plaque load, suggesting that microglia might play a role in cognitive dysfunction independent of Aβ pathology (Dagher et al., 2015). Alternatively, it has been proposed that Aβ binds to postsynaptic glutamatergic receptors leading to synapse inactivation (Decker et al., 2010; Li et al., 2011). Microglia might then be recruited to the Aβ tagged synapse and induce the removal of this complex.

## Astrocytes

Astrocytes represent the most abundant cell type in the brain. They are involved in synapse formation and elimination, synaptic plasticity and activity. Due to their essential role in brain function it is likely that astrocyte dysfunction results in progression of neurodegenerative diseases. Similar to microglia, reactive astrocytes surround senile Aβ plaques in the brain of AD patients and in mouse models of AD. They become reactive as indicated by their hypertrophic processes and increased expression of GFAP (Wisniewski and Wegiel, 1991; Sofroniew, 2009) (Figure 1B). On the one hand, astrocytes are able to degrade and phagocytose Aβ and reduce Aβ mediated neurotoxicity (Wyss-Coray et al., 2003), but on the other hand they induce microglia activation by releasing proinflammatory factors (Wyss-Coray and Rogers, 2012). Vice versa, a subtype of reactive astrocytes (A1) that is abundant in the AD brain, is induced by neuroinflammatory microglia (Liddelow et al., 2017). As AD pathology progresses, reactive astrocytes upregulate the adenosine receptor A2A, thereby leading to long-term memory loss due to affected astrocyte-synapse interactions. In addition, conditional genetic removal of the A2A-receptor enhanced memory function in hAPP mice (Orr et al., 2015). These findings suggest that increased levels of astrocytic A2A receptor due to AD pathology might contribute to memory loss. Moreover, resting Ca^2+^ levels are enhanced in AD mice and more frequent Ca^2+^ transients and intracellular Ca^2+^ waves are present, all of which can lead to the release of gliotransmitters (glutamate, ATP, GABA) (Kuchibhotla et al., 2009; Henneberger et al., 2010; Lee et al., 2010; Woo et al., 2012). Furthermore, production of GABA by reactive astrocytes is increased in APPPS1 mice, though inhibition of GABA production or release from reactive astrocytes fully recovers spike probability, synaptic plasticity, learning and memory loss in these mice (Jo et al., 2014).

Further investigations of neuron-glia signaling pathways and their disruption in neurodegenerative diseases are necessary for the development of new successful therapies that are promising due to the early involvement of glia in the disease process.

## Therapeutic Approaches

Although our knowledge regarding the mechanism underlying AD pathogenesis has improved over the last decades, there is still no cure available. Moreover, open questions concerning memory and synapse loss, as well as gliosis and related neuronal damage, still remain (De Strooper and Karran, 2016).

Most current therapeutic approaches focused on the reduction of Aβ levels and Aβ plaque load by inhibiting or modifying the generation of Aβ. Other attempts tried to target the tau protein instead (Roberson et al., 2007; Ittner et al., 2010). The reduction of endogenous WT murine tau by 50% circumvented synaptic and behavioral deficits in hAPP mice, without affecting Aβ plaque load (Roberson et al., 2007). Although the mechanism by which Aβ-mediated cognitive deficits are prevented without diminishing Aβ levels remains elusive. *In vivo* imaging of 3xTg-AD mice revealed spine loss on dystrophic dendrites positive for hyperphosphorylated tau in areas without plaques (Bittner et al., 2010). Further investigations on the function of tau in mouse models of AD will provide insights regarding the role of tau in AD.

Prime targets for AD therapies are β- and γ-secretase inhibitors. Numerous inhibitors currently undergo clinical trials (May et al., 2011; Lucas et al., 2012; Wang et al., 2014; Yan and Vassar, 2014). Therefore, several studies have tested β- and γ-secretase inhibitors in mouse models of AD. *In vivo* 2-photon imaging allows to explore structural plasticity of synapses in living mice, even for long-time periods (Grutzendler et al., 2002; Tsai et al., 2004; Spires et al., 2005; Fuhrmann et al., 2007; Liebscher and Meyer-Luehmann, 2012; Liebscher et al., 2014) and the effect of administered drugs on the plasticity of spines and synapses can be directly monitored. Two different γ-secretase inhibitors, DAPT and LY450139, were tested in WT and APP-KO mice on structural plasticity of dendritic spines. Bittner et al. (2009) could show that APP-KO mice have an increased spine density and that γ-secretase inhibition reduces the number of spines in an APP-dependent manner. Other studies performed *in vivo* 2-photon imaging and followed dendritic spines and axonal boutons over the course of several weeks in APPS1 mice. Pre- and postsynaptic structures showed an enhanced instability in the vicinity of Aβ plaques (Grutzendler and Gan, 2007; Spires-Jones et al., 2007; Liebscher et al., 2014). Four weeks treatment with a γ-secretase inhibitor (ELN594) efficiently reduced Aβ plaque formation and growth and stabilized spines near plaques (Liebscher et al., 2014).

Unfortunately, the inhibition of BACE1 is known for its mechanism-based side-effects. Conditional deletion of BACE1 in 5xFAD mice resulted in reduced Aβ plaque load and improved synaptic function, determined by LTP and contextual fear conditioning experiments (Hu et al., 2018). However, ablation of BACE1 in mice is not without issues, as those mice exhibit abnormal astrogenesis, neurogenesis, hyperactivities, impaired axonal growth and altered LTP (Vassar, 2014). Pharmacological inhibition of BACE1 slowed down plaque formation and reduced dendritic spine formation via Seizure Protein 6 in long-term *in vivo* imaging experiments (Filser et al., 2015; Peters et al., 2018; Zhu et al., 2018). Further studies are needed to elucidate how side-effects can be reduced to a minimum e.g., by partial inhibition of BACE1 (Fukumoto et al., 2002; Zhao et al., 2007).

The oligomeric form of Aβ is often considered as the toxic form. Immunotherapy against Aβ oligomers had little effect on synapse loss in the vicinity of Aβ plaques but abolished synapse loss further away from plaques (Dorostkar et al., 2014), suggesting that synapse loss is not primarily mediated by oligomers. In another study, switching off oligomer production resulted in improved cognitive and synaptic impairment (Fowler et al., 2014). However, despite these promising results in preclinical studies, removing toxic Aβ species from the brain with active immunization failed in clinical trials (Hyman, 2011).

To date, it remains an open question whether such Aβ lowering strategies will be successful. Therefore, alternative treatment options should be considered. Mice exposed to an environmental enrichment developed enhanced numbers of new dendritic spines, excitatory synapses and dendritic branches on pyramidal neurons (Mora et al., 2007). Environmental enrichment has also been shown to ameliorate Aβ plaque load, synapse loss and impaired synaptic plasticity (Lazarov et al., 2005; Cracchiolo et al., 2007; Herring et al., 2009; Ziegler-Waldkirch et al., 2018a,b). In a non-pharmacological approach, housing in an environmental enrichment reduced Aβ plaque load by activating phagocytic microglia in 5xFAD transgenic mice (Ziegler-Waldkirch et al., 2018a). Furthermore, adult neurogenesis was revived and cognitive deficits caused by induced Aβ plaque deposits were rescued (Ziegler-Waldkirch et al., 2018a). Future research on the microglia function and dysfunction in CNS disorders, such as pruning, regulating plasticity and neurogenesis will undoubtedly play a predominant role in the search for an effective cure.

## Conclusion

Besides the physical degeneration of synapses in AD and other neurodegenerative diseases, it is unclear which role glial cells play during the process of synapse loss. Further research will hopefully provide more insights into the role of glial cells and their contribution to synapse loss, in particular at earlier pre-depositing stages when synapses are already vulnerable. Future preclinical treatment approaches should combine pharmacological, non-pharmacological and behavioral studies.

## Author Contributions

SZ-W and MM-L contributed equally to this work, wrote the manuscript, read and approved the final manuscript.

## Conflict of Interest Statement

The authors declare that the research was conducted in the absence of any commercial or financial relationships that could be construed as a potential conflict of interest.

## References

[B2] Bergles D. E. Jabs R. Steinhäuser C. (2010). Neuron-glia synapses in the brain. Brain Res. Rev. 63, 130–137. 10.1016/j.brainresrev.2009.12.003 20018210 PMC2862892

[B3] Bisht K. Sharma K. P. Lecours C. Sánchez M. G. El Hajj H. Milior G.. (2016). Dark microglia: a new phenotype predominantly associated with pathological states. Glia 64, 826–839. 10.1002/glia.22966 26847266 PMC4949554

[B4] Bittner T. Fuhrmann M. Burgold S. Jung C. K. E. Volbracht C. Steiner H.. (2009). γ-secretase inhibition reduces spine density *in vivo* via an amyloid precursor protein-dependent pathway. J. Neurosci. 29, 10405–10409. 10.1523/JNEUROSCI.2288-09.2009 19692615 PMC6665795

[B5] Bittner T. Fuhrmann M. Burgold S. Ochs S. M. Hoffmann N. Mitteregger G.. (2010). Multiple events lead to dendritic spine loss in triple transgenic Alzheimer’s disease mice. PLoS One 5:e15477. 10.1371/journal.pone.0015477 21103384 PMC2982845

[B6] Bolmont T. Haiss F. Eicke D. Radde R. Mathis C. A. Klunk W. E.. (2008). Dynamics of the microglial/amyloid interaction indicate a role in plaque maintenance. J. Neurosci. 28, 4283–4292. 10.1523/JNEUROSCI.4814-07.2008 18417708 PMC3844768

[B7] Brawek B. Schwendele B. Riester K. Kohsaka S. Lerdkrai C. Liang Y.. (2014). Impairment of *in vivo* calcium signaling in amyloid plaque-associated microglia. Acta Neuropathol. 127, 495–505. 10.1007/s00401-013-1242-2 24407428

[B8] Callahan L. M. Vaules W. A. Coleman P. D. (1999). Quantitative decrease in synaptophysin message expression and increase in cathepsin D message expression in Alzheimer disease neurons containing neurofibrillary tangles. J. Neuropathol. Exp. Neurol. 58, 275–287. 10.1097/00005072-199903000-00007 10197819

[B9] Clarke L. E. Barres B. A. (2013). Emerging roles of astrocytes in neural circuit development. Nat. Rev. Neurosci. 14, 311–321. 10.1038/nrn3484 23595014 PMC4431630

[B10] Cleary J. P. Walsh D. M. Hofmeister J. J. Shankar G. M. Kuskowski M. A. Selkoe D. J.. (2005). Natural oligomers of the amyloid-β protein specifically disrupt cognitive function. Nat. Neurosci. 8, 79–84. 10.1038/nn1372 15608634

[B11] Cracchiolo J. R. Mori T. Nazian S. J. Tan J. Potter H. Arendash G. W. (2007). Enhanced cognitive activity—over and above social or physical activity—is required to protect Alzheimer’s mice against cognitive impairment, reduce Aβ deposition, and increase synaptic immunoreactivity. Neurobiol. Learn. Mem. 88, 277–294. 10.1016/j.nlm.2007.07.007 17714960 PMC2083653

[B12] Crimins J. L. Rocher A. B. Peters A. Shultz P. Lewis J. Luebke J. I. (2011). Homeostatic responses by surviving cortical pyramidal cells in neurodegenerative tauopathy. Acta Neuropathol. 122, 551–564. 10.1007/s00401-011-0877-0 21968531

[B13] D’Amore J. D. Kajdasz S. T. McLellan M. E. Bacskai B. J. Stern E. A. Hyman B. T. (2003). *In vivo* multiphoton imaging of a transgenic mouse model of Alzheimer disease reveals marked thioflavine-S-associated alterations in neurite trajectories. J. Neuropathol. Exp. Neurol. 62, 137–145. 10.1093/jnen/62.2.137 12578223

[B14] Dagher N. N. Najafi A. R. Kayala K. M. N. Elmore M. R. P. White T. E. Medeiros R.. (2015). Colony-stimulating factor 1 receptor inhibition prevents microglial plaque association and improves cognition in 3xTg-AD mice. J. Neuroinflammation 12:139. 10.1186/s12974-015-0366-9 26232154 PMC4522109

[B15] De Biase L. M. Nishiyama A. Bergles D. E. (2010). Excitability and synaptic communication within the oligodendrocyte lineage. J. Neurosci. 30, 3600–3611. 10.1523/JNEUROSCI.6000-09.2010 20219994 PMC2838193

[B16] De Strooper B. Karran E. (2016). The cellular phase of Alzheimer’s disease. Cell 164, 603–615. 10.1016/j.cell.2015.12.056 26871627

[B17] Decker H. Lo K. Y. Unger S. M. Ferreira S. T. Silverman M. A. (2010). Amyloid-β peptide oligomers disrupt axonal transport through an NMDA receptor-dependent mechanism that is mediated by glycogen synthase kinase 3β in primary cultured hippocampal neurons. J. Neurosci. 30, 9166–9171. 10.1523/JNEUROSCI.1074-10.2010 20610750 PMC6632489

[B18] Deczkowska A. Keren-Shaul H. Weiner A. Colonna M. Schwartz M. Amit I. (2018). Disease-associated microglia: a universal immune sensor of neurodegeneration. Cell 173, 1073–1081. 10.1016/j.cell.2018.05.003 29775591

[B19] Delekate A. Füchtemeier M. Schumacher T. Ulbrich C. Foddis M. Petzold G. C. (2014). Metabotropic P2Y1 receptor signalling mediates astrocytic hyperactivity *in vivo* in an Alzheimer’s disease mouse model. Nat. Commun. 5:5422. 10.1038/ncomms6422 25406732

[B20] Dimou L. Gallo V. (2015). NG2-glia and their functions in the central nervous system. Glia 63, 1429–1451. 10.1002/glia.22859 26010717 PMC4470768

[B21] Dorostkar M. M. Burgold S. Filser S. Barghorn S. Schmidt B. Anumala U. R.. (2014). Immunotherapy alleviates amyloid-associated synaptic pathology in an Alzheimer’s disease mouse model. Brain J. Neurol. 137, 3319–3326. 10.1093/brain/awu280 25281869 PMC4240293

[B22] Dudai Y. Morris R. G. M. (2013). Memorable trends. Neuron 80, 742–750. 10.1016/j.neuron.2013.09.039 24183024

[B23] Filser S. Ovsepian S. V. Masana M. Blazquez-Llorca L. Brandt Elvang A. Volbracht C.. (2015). Pharmacological inhibition of BACE1 impairs synaptic plasticity and cognitive functions. Biol. Psychiatry 77, 729–739. 10.1016/j.biopsych.2014.10.013 25599931

[B24] Fonseca M. I. Chu S. Pierce A. L. Brubaker W. D. Hauhart R. E. Mastroeni D.. (2016). Analysis of the putative role of CR1 in Alzheimer’s disease: genetic association, expression and function. PLoS One 11:e0149792. 10.1371/journal.pone.0149792 26914463 PMC4767815

[B25] Fonseca M. I. Zhou J. Botto M. Tenner A. J. (2004). Absence of C1q leads to less neuropathology in transgenic mouse models of Alzheimer’s disease. J. Neurosci. 4, 6457–6465. 10.1523/JNEUROSCI.0901-04.2004 15269255 PMC6729885

[B26] Fowler S. W. Chiang A. C. A. Savjani R. R. Larson M. E. Sherman M. A. Schuler D. R.. (2014). Genetic modulation of soluble Aβ rescues cognitive and synaptic impairment in a mouse model of Alzheimer’s disease. J. Neurosci. 34, 7871–7885. 10.1523/JNEUROSCI.4749-14.2014 24899710 PMC4044248

[B27] Freir D. B. Fedriani R. Scully D. Smith I. M. Selkoe D. J. Walsh D. M.. (2011). Aβ oligomers inhibit synapse remodelling necessary for memory consolidation. Neurobiol. Aging 32, 2211–2218. 10.1016/j.neurobiolaging.2010.01.001 20097446 PMC2891223

[B28] Fuhrmann M. Mitteregger G. Kretzschmar H. Herms J. (2007). Dendritic pathology in prion disease starts at the synaptic spine. J. Neurosci. 27, 6224–6233. 10.1523/JNEUROSCI.5062-06.2007 17553995 PMC6672160

[B29] Fukumoto H. Cheung B. S. Hyman B. T. Irizarry M. C. (2002). β-secretase protein and activity are increased in the neocortex in Alzheimer disease. Arch. Neurol. 59, 1381–1389. 10.1001/archneur.59.9.1381 12223024

[B30] Garcia-Alloza M. Dodwell S. A. Meyer-Luehmann M. Hyman B. T. Bacskai B. J. (2006). Plaque-derived oxidative stress mediates distorted neurite trajectories in the Alzheimer mouse model. J. Neuropathol. Exp. Neurol. 65, 1082–1089. 10.1097/01.jnen.0000240468.12543.af 17086105

[B31] Ginsberg S. D. Hemby S. E. Lee V. M. Eberwine J. H. Trojanowski J. Q. (2000). Expression profile of transcripts in Alzheimer’s disease tangle-bearing CA1 neurons. Ann. Neurol. 48, 77–87. 10.1002/1531-8249(200007)48:1<77::aid-ana12>3.3.co;2-1 10894219

[B32] Griciuc A. Serrano-Pozo A. Parrado A. R. Lesinski A. N. Asselin C. N. Mullin K.. (2013). Alzheimer’s disease risk gene CD33 inhibits microglial uptake of amyloid β. Neuron 78, 631–643. 10.1016/j.neuron.2013.04.014 23623698 PMC3706457

[B33] Grimaldi A. Brighi C. Peruzzi G. Ragozzino D. Bonanni V. Limatola C.. (2018). Inflammation, neurodegeneration and protein aggregation in the retina as ocular biomarkers for Alzheimer’s disease in the 3xTg-AD mouse model. Cell Death Dis. 9:685. 10.1038/s41419-018-0740-5 29880901 PMC5992214

[B34] Grundke-Iqbal I. Iqbal K. Tung Y. C. Quinlan M. Wisniewski H. M. Binder L. I. (1986). Abnormal phosphorylation of the microtubule-associated protein tau (tau) in Alzheimer cytoskeletal pathology. Proc. Natl. Acad. Sci. U S A 83, 4913–4917. 10.1073/pnas.83.13.4913 3088567 PMC323854

[B35] Grutzendler J. Gan W.-B. (2007). Long-term two-photon transcranial imaging of synaptic structures in the living brain. CSH Protoc. 2007:pdb.prot4766. 10.1101/pdb.prot4766 21357119

[B36] Grutzendler J. Kasthuri N. Gan W.-B. (2002). Long-term dendritic spine stability in the adult cortex. Nature 420, 812–816. 10.1038/nature01276 12490949

[B37] Guerreiro R. Wojtas A. Bras J. Carrasquillo M. Rogaeva E. Majounie E.. (2013). TREM2 variants in Alzheimer’s disease. N. Engl. J. Med. 368, 117–127. 10.1056/NEJMoa1211851 23150934 PMC3631573

[B38] Haass C. (2004). Take five—BACE and the γ-secretase quartet conduct Alzheimer’s amyloid β-peptide generation. EMBO J. 23, 483–488. 10.1038/sj.emboj.7600061 14749724 PMC1271800

[B39] Harold D. Abraham R. Hollingworth P. Sims R. Gerrish A. Hamshere M. L.. (2009). Genome-wide association study identifies variants at CLU and PICALM associated with Alzheimer’s disease. Nat. Genet. 41, 1088–1093. 10.1038/ng.440 19734902 PMC2845877

[B40] Harper J. D. Lansbury P. T. Jr. (1997). Models of amyloid seeding in Alzheimer’s disease and scrapie: mechanistic truths and physiological consequences of the time-dependent solubility of amyloid proteins. Annu. Rev. Biochem. 66, 385–407. 10.1146/annurev.biochem.66.1.385 9242912

[B41] Harris J. A. Devidze N. Halabisky B. Lo I. Thwin M. T. Yu G.-Q.. (2010). Many neuronal and behavioral impairments in transgenic mouse models of Alzheimer’s disease are independent of caspase cleavage of the amyloid precursor protein. J. Neurosci. 30, 372–381. 10.1523/JNEUROSCI.5341-09.2010 20053918 PMC3064502

[B42] Henneberger C. Papouin T. Oliet S. H. R. Rusakov D. A. (2010). Long-term potentiation depends on release of D-serine from astrocytes. Nature 463, 232–236. 10.1038/nature08673 20075918 PMC2807667

[B43] Herring A. Ambrée O. Tomm M. Habermann H. Sachser N. Paulus W.. (2009). Environmental enrichment enhances cellular plasticity in transgenic mice with Alzheimer-like pathology. Exp. Neurol. 216, 184–192. 10.1016/j.expneurol.2008.11.027 19118549

[B44] Hollingworth P. Sweet R. Sims R. Harold D. Russo G. Abraham R.. (2012). Genome-wide association study of Alzheimer’s disease with psychotic symptoms. Mol. Psychiatry 17, 1316–1327. 10.1038/mp.2011.125 22005930 PMC3272435

[B45] Hong S. Beja-Glasser V. F. Nfonoyim B. M. Frouin A. Li S. Ramakrishnan S.. (2016). Complement and microglia mediate early synapse loss in Alzheimer mouse models. Science 352, 712–716. 10.1126/science.aad8373 27033548 PMC5094372

[B46] Hsia A. Y. Masliah E. McConlogue L. Yu G. Q. Tatsuno G. Hu K.. (1999). Plaque-independent disruption of neural circuits in Alzheimer’s disease mouse models. Proc. Natl. Acad. Sci. U S A 96, 3228–3233. 10.1073/pnas.96.6.3228 10077666 PMC15924

[B47] Hu X. Das B. Hou H. He W. Yan R. (2018). BACE1 deletion in the adult mouse reverses preformed amyloid deposition and improves cognitive functions. J. Exp. Med. 215, 927–940. 10.1084/jem.20171831 29444819 PMC5839766

[B48] Hyman B. T. (2011). Amyloid-dependent and amyloid-independent stages of Alzheimer disease. Arch. Neurol. 68, 1062–1064. 10.1001/archneurol.2011.70 21482918

[B49] Ittner L. M. Ke Y. D. Delerue F. Bi M. Gladbach A. van Eersel J.. (2010). Dendritic function of tau mediates amyloid-β toxicity in Alzheimer’s disease mouse models. Cell 142, 387–397. 10.1016/j.cell.2010.06.036 20655099

[B50] Jo S. Yarishkin O. Hwang Y. J. Chun Y. E. Park M. Woo D. H.. (2014). GABA from reactive astrocytes impairs memory in mouse models of Alzheimer’s disease. Nat. Med. 20, 886–896. 10.1038/nm.3639 24973918 PMC8385452

[B51] Jonsson T. Stefansson H. Steinberg S. Jonsdottir I. Jonsson P. V. Snaedal J.. (2013). Variant of TREM2 associated with the risk of Alzheimer’s disease. N. Engl. J. Med. 368, 107–116. 10.1056/NEJMoa1211103 23150908 PMC3677583

[B52] Jun G. Naj A. C. Beecham G. W. Wang L.-S. Buros J. Gallins P. J.. (2010). Meta-analysis confirms CR1, CLU, and PICALM as alzheimer disease risk loci and reveals interactions with APOE genotypes. Arch. Neurol. 67, 1473–1484. 10.1001/archneurol.2010.201 20697030 PMC3048805

[B53] Keren-Shaul H. Spinrad A. Weiner A. Matcovitch-Natan O. Dvir-Szternfeld R. Ulland T. K.. (2017). A unique microglia type associated with restricting development of Alzheimer’s disease. Cell 169, 1276–1290. 10.1016/j.cell.2017.05.018 28602351

[B54] Koffie R. M. Hyman B. T. Spires-Jones T. L. (2011). Alzheimer’s disease: synapses gone cold. Mol. Neurodegener. 6:63. 10.1186/1750-1326-6-63 21871088 PMC3178498

[B55] Koffie R. M. Meyer-Luehmann M. Hashimoto T. Adams K. W. Mielke M. L. Garcia-Alloza M.. (2009). Oligomeric amyloid β associates with postsynaptic densities and correlates with excitatory synapse loss near senile plaques. Proc. Natl. Acad. Sci. U S A 106, 4012–4017. 10.1073/pnas.0811698106 19228947 PMC2656196

[B56] Krasemann S. Madore C. Cialic R. Baufeld C. Calcagno N. El Fatimy R.. (2017). The TREM2-APOE pathway drives the transcriptional phenotype of dysfunctional microglia in neurodegenerative diseases. Immunity 47, 566.e9–581.e9. 10.1016/j.immuni.2017.08.008 28930663 PMC5719893

[B57] Kuchibhotla K. V. Lattarulo C. R. Hyman B. T. Bacskai B. J. (2009). Synchronous hyperactivity and intercellular calcium waves in astrocytes in Alzheimer mice. Science 323, 1211–1215. 10.1126/science.1169096 19251629 PMC2884172

[B58] Kumar S. Walter J. (2011). Phosphorylation of amyloid β (Aβ) peptides—a trigger for formation of toxic aggregates in Alzheimer’s disease. Aging 3, 803–812. 10.18632/aging.100362 21869458 PMC3184981

[B59] Lambert J.-C. Heath S. Even G. Campion D. Sleegers K. Hiltunen M.. (2009). Genome-wide association study identifies variants at CLU and CR1 associated with Alzheimer’s disease. Nat. Genet. 41, 1094–1099. 10.1038/ng.439 19734903

[B60] Lazarov O. Robinson J. Tang Y.-P. Hairston I. S. Korade-Mirnics Z. Lee V. M.-Y.. (2005). Environmental enrichment reduces Aβ levels and amyloid deposition in transgenic mice. Cell 120, 701–713. 10.1016/j.cell.2005.01.015 15766532

[B61] Lee S. Yoon B.-E. Berglund K. Oh S.-J. Park H. Shin H.-S.. (2010). Channel-mediated tonic GABA release from glia. Science 330, 790–796. 10.1126/science.1184334 20929730

[B62] Lesné S. Koh M. T. Kotilinek L. Kayed R. Glabe C. G. Yang A.. (2006). A specific amyloid-β protein assembly in the brain impairs memory. Nature 440, 352–357. 10.1038/nature04533 16541076

[B63] Li S. Hong S. Shepardson N. E. Walsh D. M. Shankar G. M. Selkoe D. (2009). Soluble oligomers of amyloid β protein facilitate hippocampal long-term depression by disrupting neuronal glutamate uptake. Neuron 62, 788–801. 10.1016/j.neuron.2009.05.012 19555648 PMC2702854

[B64] Li S. Jin M. Koeglsperger T. Shepardson N. E. Shankar G. M. Selkoe D. J. (2011). Soluble Aβ oligomers inhibit long-term potentiation through a mechanism involving excessive activation of extrasynaptic NR2B-containing NMDA receptors. J. Neurosci. 31, 6627–6638. 10.1523/JNEUROSCI.0203-11.2011 21543591 PMC3100898

[B65] Liddelow S. A. Guttenplan K. A. Clarke L. E. Bennett F. C. Bohlen C. J. Schirmer L.. (2017). Neurotoxic reactive astrocytes are induced by activated microglia. Nature 541, 481–487. 10.1038/nature21029 28099414 PMC5404890

[B66] Liebscher S. Meyer-Luehmann M. (2012). A peephole into the brain: neuropathological features of Alzheimer’s disease revealed by *in vivo* two-photon imaging. Front. Psychiatry 3:26. 10.3389/fpsyt.2012.00026 22485096 PMC3317174

[B67] Liebscher S. Page R. M. Käfer K. Winkler E. Quinn K. Goldbach E.. (2014). Chronic γ-secretase inhibition reduces amyloid plaque-associated instability of pre- and postsynaptic structures. Mol. Psychiatry 19, 937–946. 10.1038/mp.2013.122 24061497 PMC4113951

[B68] Lin S.-C. Bergles D. E. (2004). Synaptic signaling between neurons and glia. Glia 47, 290–298. 10.1002/glia.20060 15252819

[B69] Lucas F. Fukushima T. Nozaki Y. (2012). Novel BACE1 inhibitor, E2609, lowers Aβ levels in the cerebrospinal fluid and plasma in nonhuman primates. Alzheimers Dement. 8:P224 10.1016/j.jalz.2012.05.2022

[B70] Maletic-Savatic M. Malinow R. Svoboda K. (1999). Rapid dendritic morphogenesis in CA1 hippocampal dendrites induced by synaptic activity. Science 283, 1923–1927. 10.1126/science.283.5409.1923 10082466

[B71] Masliah E. (2001). Recent advances in the understanding of the role of synaptic proteins in Alzheimer’s disease and other neurodegenerative disorders. J. Alzheimers Dis. 3, 121–129. 10.3233/jad-2001-3117 12214081

[B72] Masliah E. Mallory M. Hansen L. DeTeresa R. Alford M. Terry R. (1994). Synaptic and neuritic alterations during the progression of Alzheimer’s disease. Neurosci. Lett. 174, 67–72. 10.1016/0304-3940(94)90121-x 7970158

[B73] Masliah E. Terry R. D. Mallory M. Alford M. Hansen L. A. (1990). Diffuse plaques do not accentuate synapse loss in Alzheimer’s disease. Am. J. Pathol. 137, 1293–1297. 2124413 PMC1877714

[B74] May P. C. Dean R. A. Lowe S. L. Martenyi F. Sheehan S. M. Boggs L. N.. (2011). Robust central reduction of amyloid-β in humans with an orally available, non-peptidic β-secretase inhibitor. J. Neurosci. 31, 16507–16516. 10.1523/JNEUROSCI.3647-11.2011 22090477 PMC6633289

[B75] McCarter J. F. Liebscher S. Bachhuber T. Abou-Ajram C. Hübener M. Hyman B. T.. (2013). Clustering of plaques contributes to plaque growth in a mouse model of Alzheimer’s disease. Acta Neuropathol. 126, 179–188. 10.1007/s00401-013-1137-2 23775142 PMC3722456

[B76] Meyer-Luehmann M. Mielke M. Spires-Jones T. L. Stoothoff W. Jones P. Bacskai B. J.. (2009). A reporter of local dendritic translocation shows plaque- related loss of neural system function in APP-transgenic mice. J. Neurosci. 29, 12636–12640. 10.1523/JNEUROSCI.1948-09.2009 19812338 PMC2789808

[B77] Meyer-Luehmann M. Spires-Jones T. L. Prada C. Garcia-Alloza M. de Calignon A. Rozkalne A.. (2008). Rapid appearance and local toxicity of amyloid-β plaques in a mouse model of Alzheimer’s disease. Nature 451, 720–724. 10.1038/nature06616 18256671 PMC3264491

[B78] Mora F. Segovia G. del Arco A. (2007). Aging, plasticity and environmental enrichment: structural changes and neurotransmitter dynamics in several areas of the brain. Brain Res. Rev. 55, 78–88. 10.1016/j.brainresrev.2007.03.011 17561265

[B79] Mucke L. Masliah E. Yu G.-Q. Mallory M. Rockenstein E. M. Tatsuno G.. (2000). High-level neuronal expression of aβ_1–42_ in wild-type human amyloid protein precursor transgenic mice: synaptotoxicity without plaque formation. J. Neurosci. 20, 4050–4058. 10.1523/JNEUROSCI.20-11-04050.2000 10818140 PMC6772621

[B80] Nägerl U. V. Eberhorn N. Cambridge S. B. Bonhoeffer T. (2004). Bidirectional activity-dependent morphological plasticity in hippocampal neurons. Neuron 44, 759–767. 10.1016/j.neuron.2004.11.016 15572108

[B81] Naj A. C. Jun G. Beecham G. W. Wang L.-S. Vardarajan B. N. Buros J.. (2011). Common variants at MS4A4/MS4A6E, CD2AP, CD33 and EPHA1 are associated with late-onset Alzheimer’s disease. Nat. Genet. 43, 436–441. 10.1038/ng.801 21460841 PMC3090745

[B82] Nimmerjahn A. Kirchhoff F. Helmchen F. (2005). Resting microglial cells are highly dynamic surveillants of brain parenchyma *in vivo*. Science 308, 1314–1318. 10.1126/science.1110647 15831717

[B83] Ning A. Cui J. To E. Ashe K. H. Matsubara J. (2008). Amyloid-β deposits lead to retinal degeneration in a mouse model of Alzheimer disease. Invest. Ophthalmol. Vis. Sci. 49, 5136–5143. 10.1167/iovs.08-1849 18566467 PMC3947384

[B84] Orr A. G. Hsiao E. C. Wang M. M. Ho K. Kim D. H. Wang X.. (2015). Astrocytic adenosine receptor A_2A_ and G_s_-coupled signaling regulate memory. Nat. Neurosci. 18, 423–434. 10.1038/nn.3930 25622143 PMC4340760

[B85] Paolicelli R. C. Bolasco G. Pagani F. Maggi L. Scianni M. Panzanelli P.. (2011). Synaptic pruning by microglia is necessary for normal brain development. Science 333, 1456–1458. 10.1126/science.1202529 21778362

[B86] Parkhurst C. N. Yang G. Ninan I. Savas J. N. Yates J. R. III. Lafaille J. J.. (2013). Microglia promote learning-dependent synapse formation through brain-derived neurotrophic factor. Cell 155, 1596–1609. 10.1016/j.cell.2013.11.030 24360280 PMC4033691

[B87] Peters F. Salihoglu H. Rodrigues E. Herzog E. Blume T. Filser S.. (2018). BACE1 inhibition more effectively suppresses initiation than progression of β-amyloid pathology. Acta Neuropathol. 135, 695–710. 10.1007/s00401-017-1804-9 29327084 PMC5904228

[B88] Prince M. J. (2015). World Alzheimer Report 2015: The Global Impact of Dementia. Available online at: https://www.alz.co.uk/research/world-report-2015 [Accessed on August 29, 2018].

[B89] Roberson E. D. Scearce-Levie K. Palop J. J. Yan F. Cheng I. H. Wu T.. (2007). Reducing endogenous tau ameliorates amyloid β-induced deficits in an Alzheimer’s disease mouse model. Science 316, 750–754. 10.1126/science.1141736 17478722

[B90] Rogers J. Schultz J. Brachova L. Lue L. F. Webster S. Bradt B.. (1992). Complement activation and β-amyloid-mediated neurotoxicity in Alzheimer’s disease. Res. Immunol. 143, 624–630. 1455054 10.1016/0923-2494(92)80046-n

[B91] Schafer D. P. Lehrman E. K. Kautzman A. G. Koyama R. Mardinly A. R. Yamasaki R.. (2012). Microglia sculpt postnatal neural circuits in an activity and complement-dependent manner. Neuron 74, 691–705. 10.1016/j.neuron.2012.03.026 22632727 PMC3528177

[B92] Scheff S. W. DeKosky S. T. Price D. A. (1990). Quantitative assessment of cortical synaptic density in Alzheimer’s disease. Neurobiol. Aging 11, 29–37. 10.1016/0197-4580(90)90059-9 2325814

[B93] Serrano-Pozo A. Frosch M. P. Masliah E. Hyman B. T. (2011). Neuropathological alterations in Alzheimer disease. Cold Spring Harb. Perspect. Med. 1:a006189. 10.1101/cshperspect.a006189 22229116 PMC3234452

[B94] Serrano-Pozo A. Muzikansky A. Gómez-Isla T. Growdon J. H. Betensky R. A. Frosch M. P.. (2013). Differential relationships of reactive astrocytes and microglia to fibrillar amyloid deposits in Alzheimer disease. J. Neuropathol. Exp. Neurol. 72, 462–471. 10.1097/nen.0b013e3182933788 23656989 PMC3661683

[B95] Shankar G. M. Bloodgood B. L. Townsend M. Walsh D. M. Selkoe D. J. Sabatini B. L. (2007). Natural oligomers of the Alzheimer amyloid-β protein induce reversible synapse loss by modulating an NMDA-type glutamate receptor-dependent signaling pathway. J. Neurosci. 27, 2866–2875. 10.1523/JNEUROSCI.4970-06.2007 17360908 PMC6672572

[B96] Shankar G. M. Leissring M. A. Adame A. Sun X. Spooner E. Masliah E.. (2009). Biochemical and immunohistochemical analysis of an Alzheimer’s disease mouse model reveals the presence of multiple cerebral Aβ assembly forms throughout life. Neurobiol. Dis. 36, 293–302. 10.1016/j.nbd.2009.07.021 19660551 PMC2782414

[B97] Shankar G. M. Li S. Mehta T. H. Garcia-Munoz A. Shepardson N. E. Smith I.. (2008). Amyloid-β protein dimers isolated directly from Alzheimer’s brains impair synaptic plasticity and memory. Nat. Med. 14, 837–842. 10.1038/nm1782 18568035 PMC2772133

[B98] Shen Y. Lue L. Yang L. Roher A. Kuo Y. Strohmeyer R.. (2001). Complement activation by neurofibrillary tangles in Alzheimer’s disease. Neurosci. Lett. 305, 165–168. 10.1016/S0304-3940(01)01842-0 11403931

[B99] Sofroniew M. V. (2009). Molecular dissection of reactive astrogliosis and glial scar formation. Trends Neurosci. 32, 638–647. 10.1016/j.tins.2009.08.002 19782411 PMC2787735

[B100] Spires T. L. Meyer-Luehmann M. Stern E. A. McLean P. J. Skoch J. Nguyen P. T.. (2005). Dendritic spine abnormalities in amyloid precursor protein transgenic mice demonstrated by gene transfer and intravital multiphoton microscopy. J. Neurosci. 25, 7278–7287. 10.1523/JNEUROSCI.1879-05.2005 16079410 PMC1820616

[B101] Spires-Jones T. L. Meyer-Luehmann M. Osetek J. D. Jones P. B. Stern E. A. Bacskai B. J.. (2007). Impaired spine stability underlies plaque-related spine loss in an Alzheimer’s disease mouse model. Am. J. Pathol. 171, 1304–1311. 10.2353/ajpath.2007.070055 17717139 PMC1988879

[B102] Spires-Jones T. L. Stoothoff W. H. de Calignon A. Jones P. B. Hyman B. T. (2009). Tau pathophysiology in neurodegeneration: a tangled issue. Trends Neurosci. 32, 150–159. 10.1016/j.tins.2008.11.007 19162340

[B1] Stelzmann R. A. Schnitzlein H. N. Murtagh F. R. (1995). An English translation of Alzheimer’s 1907 paper, “Über eine eigenartige Erkankung der Hirnrinde”. Clin. Anat. 8, 429–431. 10.1002/ca.980080612 8713166

[B103] Stephan A. H. Barres B. A. Stevens B. (2012). The complement system: an unexpected role in synaptic pruning during development and disease. Annu. Rev. Neurosci. 35, 369–389. 10.1146/annurev-neuro-061010-113810 22715882

[B104] Stevens B. Allen N. J. Vazquez L. E. Howell G. R. Christopherson K. S. Nouri N.. (2007). The classical complement cascade mediates CNS synapse elimination. Cell 131, 1164–1178. 10.1016/j.cell.2007.10.036 18083105

[B105] Takahashi R. H. Almeida C. G. Kearney P. F. Yu F. Lin M. T. Milner T. A.. (2004). Oligomerization of Alzheimer’s β-amyloid within processes and synapses of cultured neurons and brain. J. Neurosci. 24, 3592–3599. 10.1523/JNEUROSCI.5167-03.2004 15071107 PMC6729733

[B106] Terry R. D. Masliah E. Salmon D. P. Butters N. DeTeresa R. Hill R.. (1991). Physical basis of cognitive alterations in Alzheimer’s disease: synapse loss is the major correlate of cognitive impairment. Ann. Neurol. 30, 572–580. 10.1002/ana.410300410 1789684

[B107] Tremblay M.-E. Riad M. Majewska A. (2010). Preparation of mouse brain tissue for immunoelectron microscopy. J. Vis. Exp. 41:e2021. 10.3791/2021 20689505 PMC3156065

[B108] Tsai J. Grutzendler J. Duff K. Gan W.-B. (2004). Fibrillar amyloid deposition leads to local synaptic abnormalities and breakage of neuronal branches. Nat. Neurosci. 7, 1181–1183. 10.1038/nn1335 15475950

[B109] Vassar R. (2014). BACE1 inhibitor drugs in clinical trials for Alzheimer’s disease. Alzheimers Res. Ther. 6:89. 10.1186/s13195-014-0089-7 25621019 PMC4304279

[B110] Verkhratsky A. Olabarria M. Noristani H. N. Yeh C.-Y. Rodriguez J. J. (2010). Astrocytes in Alzheimer’s disease. Neurother. J. Am. Soc. Exp. Neurother. 7, 399–412. 10.1016/j.nurt.2010.05.017 20880504 PMC5084302

[B111] Walsh D. M. Klyubin I. Fadeeva J. V. Cullen W. K. Anwyl R. Wolfe M. S.. (2002). Naturally secreted oligomers of amyloid β protein potently inhibit hippocampal long-term potentiation *in vivo*. Nature 416, 535–539. 10.1038/416535a 11932745

[B112] Wang H. Megill A. Wong P. C. Kirkwood A. Lee H.-K. (2014). Postsynaptic target specific synaptic dysfunctions in the CA3 area of BACE1 knockout mice. PLoS One 9:e92279. 10.1371/journal.pone.0092279 24637500 PMC3956924

[B113] Wisniewski H. M. Wegiel J. (1991). Spatial relationships between astrocytes and classical plaque components. Neurobiol. Aging 12, 593–600. 10.1016/0197-4580(91)90091-w 1770990

[B114] Woo D. H. Han K.-S. Shim J. W. Yoon B.-E. Kim E. Bae J. Y.. (2012). TREK-1 and Best1 channels mediate fast and slow glutamate release in astrocytes upon GPCR activation. Cell 151, 25–40. 10.1016/j.cell.2012.09.005 23021213

[B115] Wu Y. Dissing-Olesen L. MacVicar B. A. Stevens B. (2015). Microglia: dynamic mediators of synapse development and plasticity. Trends Immunol. 36, 605–613. 10.1016/j.it.2015.08.008 26431938 PMC4841266

[B117] Wyss-Coray T. Loike J. D. Brionne T. C. Lu E. Anankov R. Yan F.. (2003). Adult mouse astrocytes degrade amyloid-β *in vitro* and *in situ*. Nat. Med. 9, 453–457. 10.1038/nm838 12612547

[B116] Wyss-Coray T. Rogers J. (2012). Inflammation in Alzheimer disease-a brief review of the basic science and clinical literature. Cold Spring Harb. Perspect. Med. 2:a006346. 10.1101/cshperspect.a006346 22315714 PMC3253025

[B118] Yan R. Vassar R. (2014). Targeting the β secretase BACE1 for Alzheimer’s disease therapy. Lancet Neurol. 13, 319–329. 10.1016/S1474-4422(13)70276-X 24556009 PMC4086426

[B119] Yang T. Li S. Xu H. Walsh D. M. Selkoe D. J. (2017). Large soluble oligomers of amyloid β-protein from Alzheimer brain are far less neuroactive than the smaller oligomers to which they dissociate. J. Neurosci. 37, 152–163. 10.1523/JNEUROSCI.1698-16.2016 28053038 PMC5214627

[B120] Zhan Y. Paolicelli R. C. Sforazzini F. Weinhard L. Bolasco G. Pagani F.. (2014). Deficient neuron-microglia signaling results in impaired functional brain connectivity and social behavior. Nat. Neurosci. 17, 400–406. 10.1038/nn.3641 24487234

[B121] Zhao J. Fu Y. Yasvoina M. Shao P. Hitt B. O’Connor T.. (2007). β-site amyloid precursor protein cleaving enzyme 1 levels become elevated in neurons around amyloid plaques: implications for Alzheimer’s disease pathogenesis. J. Neurosci. 27, 3639–3649. 10.1523/JNEUROSCI.4396-06.2007 17409228 PMC6672403

[B122] Zhu K. Peters F. Filser S. Herms J. (2018). Consequences of pharmacological BACE inhibition on synaptic structure and function. Biol. Psychiatry 84, 478–487. 10.1016/j.biopsych.2018.04.022 29945719

[B123] Ziegler-Waldkirch S. d’Errico P. Sauer J.-F. Erny D. Savanthrapadian S. Loreth D.. (2018a). Seed-induced Aβ deposition is modulated by microglia under environmental enrichment in a mouse model of Alzheimer’s disease. EMBO J. 37, 167–182. 10.15252/embj.201797021 29229786 PMC5770788

[B124] Ziegler-Waldkirch S. Marksteiner K. Stoll J. d’Errico P. Friesen M. Eiler D.. (2018b). Environmental enrichment reverses Aβ pathology during pregnancy in a mouse model of Alzheimer’s disease. Acta Neuropathol. Commun. 6:44. 10.1186/s40478-018-0549-6 29855361 PMC5984325

